# Water at Meals

**Published:** 1891-02

**Authors:** 


### WATER AT MEALS.

Opinions differ as to the effect of the free ingestion of water at meal-times, but the view most generally received is probably that it dilutes the gastric juice and so retards digestion. Apart from the fact that a moderate delay in the process is by no means a disadvantage, it is more than doubtful whether any such effect is in reality produced. When ingested during meals, water may do good by washing out the digested food and by exposing the undigested part more thoroughly to the action of the digestive ferments. The good effect of water drunk freely before meals has, however, another beneficial result—it washes away the mucus which is secreted by the mucous membrane during the intervals of repose, and favors peristalsis of the whole alimentary tract.

The membrane thus cleansed is in a much better condition to receive food and convert it into soluble compounds. Exercise before partaking of a meal stimulates the circulation of the blood, washes out the mucus, partially distends the stomach, wakes up peristalsis, and prepares the alimentary canal for the morning meal. Observation has shown that non-irritating liquids pass through the "tubular" stomach, and even if food be present, they only mix with it to a slight extent. According to Dr. Leuf, who has made this subject a special study, cold water should be given to persons who have sufficient vitality to react, and hot water to others. In chronic gastric catarrh it is extremely beneficial to drink warm or hot water before meals.—*British Medical Journal.*

